# Supplementary material for: Analysis of PPARγ Signaling Activity in Psoriasis
Source: Int J Mol Sci. 2021 Aug 10;22(16):8603. doi: 10.3390/ijms22168603 (PMC8395241; doi:10.3390/ijms22168603)
Supplement: Supplementary file 1 [file ijms-22-08603-s001.zip › Supplemental materials_Analysis of PPARg signaling activity in psoriasis/Pathway models/Models images and html files/PPARG expressed in cells assotiated with psoriasis/225675.html]

cytotoxic T-cell


# CellType cytotoxic T-cell

|  |  |
| --- | --- |
| URN | urn:agi-ncimcelltype:C0039195 |
| Total Entities | 47 |
| Connectivity | 3282 |
| Name | cytotoxic T-cell |

---

|  |  |
| --- | --- |
| ChildConcepts | NK cell mediated cytotoxicity |
|  | cytotoxic T-cell differentiation |
|  | antigen specific cytotoxic T-cell |
|  | CD4+ cytotoxic T-cell |
|  | Tc1 cell |
|  | Tc2 cell |
|  | cytotoxic T-cell infiltration |
|  | cytotoxic T-cell growth |
|  | cytotoxic T-cell mediated cytotoxicity |
|  | cytotoxic T-cell population |
|  | cytotoxic T-cell proliferation |
|  | cytotoxic T-cell development |
|  | cytotoxic T-cell survival |
|  | cytotoxic T-cell death |
|  | cytotoxic T-cell formation |
|  | cytotoxic T-cell cytotoxicity |
|  | cytotoxic T-cell homing |
|  | cytotoxic T-cell apoptosis |
|  | cytotoxic T-cell count |
|  | cytotoxic T-cell priming |
|  | cytotoxic T-cell migration |
|  | cytotoxic T-cell recognition |
|  | cytotoxic T-cell damage |
|  | cytotoxic T-cell adhesion |
|  | cytotoxic T-cell polarity |
|  | cytotoxic T-cell motility |
|  | cytotoxic T-cell homeostasis |
|  | cytotoxic T-cell repertoire |
|  | cytotoxic T-cell chemotaxis |
|  | cytotoxic T-cell destruction |
|  | cytotoxic T-cell phenotype |
|  | cytotoxic T-cell interaction |
|  | cytotoxic T-cell clonal anergy |
|  | cytotoxic T-cell localization |
|  | cytotoxic T-cell extravasation |
|  | cytotoxic T-cell ratio |
|  | cytotoxic T-cell transendothelial migration |
|  | cytotoxic T-cell distribution |
|  | cytotoxic T-cell proliferative response |
|  | cytotoxic T-cell viability |
|  | cytotoxic T-cell invasion |
|  | cytotoxic T-cell phagocytosis |
|  | cytotoxic T-cell sequestration |
|  | cytotoxic T-cell selection |
|  | cytotoxic T-cell communication |
|  | cytotoxic T-cell diapedesis |
|  | cytotoxic T-cell size |

---

|  |  |
| --- | --- |
| Pathway | Immunoglobulin Superfamily -> Expression Targets in Lymphoid System and Blood |
|  | PPARG expressed in cells assotiated with psoriasis |

---

|  |  |
| --- | --- |
| MedScan ID | 10004131 |

---

|  |  |
| --- | --- |
| Alias | killer T cells |
|  | killer T cell |
|  | T-Lymphocyte, Cytotoxic |
|  | cytotoxic T-cell |
|  | cytolytic T-lymphocyte |
|  | cytotoxic T-Lymphocytes |
|  | Alpha/Beta Cells of Cytotoxic Type |
|  | cytotoxic T-cells |
|  | Tc cell |
|  | T-Lymphocytes with a Cytotoxic Phenotype |
|  | T-Lymphocytes, Cytotoxic |
|  | T-killer cells |
|  | T-Lymphocyte with a Cytotoxic Phenotype |
|  | cytotoxic T-Lymphocyte |
|  | T-killer cell |
|  | cytolytic T-lymphocytes |
|  | Alpha/Beta Cell of Cytotoxic Type |
|  | Gamma/Delta Cell of Cytotoxic Type |
|  | Tc cells |
|  | Gamma/Delta Cells of Cytotoxic Type |

---

|  |  |
| --- | --- |
| NCIm ID | C0039195 |

---
